# Respiratory syncytial virus: impact of the disease and preventive strategies in pregnant women and older adults: Number 6 – 2024

**DOI:** 10.61622/rbgo/2024FPS06

**Published:** 2024-05-27

**Authors:** Cecília Maria Roteli-Martins, Isabela de Assis Martins Ballalai, Renato de Ávila Kfouri, Susana Cristina Aidé Viviani Fialho

**Affiliations:** Faculdade de Medicina do ABC Santo André SP Brazil Faculdade de Medicina do ABC, Santo André, SP, Brazil.; Sociedade Brasileira de Imunizações São Paulo SP Brazil Sociedade Brasileira de Imunizações, São Paulo, SP, Brazil.; Departamento de Imunizações da Sociedade Brasileira de Pediatria (SBP) Curitiba PR Brazil Departamento de Imunizações da Sociedade Brasileira de Pediatria (SBP), Curitiba, PR, Brazil.; Universidade Federal Fluminense Niterói RJ Brazil Universidade Federal Fluminense, Niterói, RJ, Brazil.

## Key points

- Address the epidemiological aspects of respiratory syncytial virus (RSV) infection and the negative impact of infections on newborns and older adults.
- Inform about the negative impacts on the health of pregnant and older adult women of respiratory infections caused by RSV and its complications.
- Bring knowledge of the evidence from studies on RSV vaccines in adult, pregnant and older adult women for a shared decision making between professionals and their patients.
- Clarify the current approval stage of new RSV prevention and treatment tools for updated clinical practice.
- Update gynecologists and obstetricians on new opportunities for active and passive immunization against RSV and on the position of Febrasgo regarding products approved for use in Brazil.

## Recommendations

- Febrasgo understands the negative impact of RSV infections on newborns and older adults, and recommends that healthcare professionals be aware of respiratory manifestations caused by this pathogen, with special attention to the group of pregnant and older adult women.
- Febrasgo has the role of bringing knowledge of the evidence from studies on RSV vaccines in adult, pregnant and older adult women for a future shared decision between professionals and their patients.
- Febrasgo monitors the published evidence on RSV vaccines and awaits availability in our country to advance specific recommendations with this further possibility of prevention in older adult and pregnant women and consequently, in newborns.
- Febrasgo believes the best way to contain the large volume of misinformation about vaccines currently occurring on social media is to offer gynecologists and obstetricians a constant update with the best scientific evidence available.
- The Specialized National Commission on Vaccines of Febrasgo understands the need to take advantage of every opportunity to inform and update health professionals about the benefits and risks of vaccines and vaccine-preventable diseases. This way they can make the necessary choices and recommendations for their patients, thereby reducing deaths, hospitalizations and negative impacts on families and health systems.
- Febrasgo believes the necessary and correct information might help to reduce vaccine hesitancy as well as morbidity and mortality for pathogens, especially those that affect individuals at the extremes of life, such as RSV.

## Background

The incubation period for RSV infection varies from two to eight days from the onset of symptoms. The transmissibility period begins 48 hours before the onset of symptoms and lasts until clinical improvement. Transmission occurs through direct contact with respiratory secretions from infected people, or indirectly, through contaminated objects and surfaces where the virus can survive for up to 24 hours. The virus enters the new host's body through the mucous membranes of the mouth, nose or eyes and also through the inhalation of droplets eliminated by the infected person's cough or sneeze. Clinical manifestations of RSV infection vary from mild or even asymptomatic forms to severe involvement of the lower respiratory tract with risk of death. The main radiological findings of RSV infection are characterized by interstitial infiltrates, hyperinflation and atelectasis. Specific diagnosis of RSV is made by using viral isolation methods, detection of viral antigens (point of care rapid detection tests and direct immunofluorescence tests) or molecular tests.^(1)^

## What is the epidemiology of diseases caused by RSV infection?

The RSV was first identified in 1955 in chimpanzees and shortly after, recognized as a cause of respiratory disease in children, especially acute viral bronchiolitis.^(1)^ Since then, this pathogen has stood out on the world stage as an important cause of acute respiratory infections (ARIs) in infants and children aged under 5 years, with a higher concentration of severe cases among young infants under 6 months.^(1,2)^

The first infection generally occurs before the age of 2, and reinfections occur throughout life, since natural infection with RSV, like other viral agents of the respiratory tract, does not induce lasting immunity (incomplete immune response). The younger the child the greater the chance of complications, hospitalizations and death. Furthermore, there is evidence of the important role of RSV infections in the development of severe forms in older adults due to the progressive age-related decline in immune function, known as immunosenescence. Likewise, there is a greater risk of unfavorable outcomes among adults with chronic diseases, such as cardiopulmonary diseases (chronic obstructive pulmonary disease [COPD], asthma, congestive heart failure, coronary artery disease and cerebrovascular disease), diabetes mellitus and chronic kidney disease, and those immunocompromised by disease or treatment.^(1–4)^ Several studies suggest that the burden of disease associated with RSV infections in the adult population, especially in older adults, is greater than the morbidity caused by the different strains of the influenza virus.^(1)^

The RSV has a significant negative impact on the health of adults. It is the third most prevalent virus in respiratory infections in older adults and responsible for exacerbations of chronic respiratory pathologies, with morbidity and mortality rates that may be higher than influenza rates in this population.^(5)^ A recent meta-analysis estimated that in 2019, 5.2 million cases of RSV among adults aged 60 years or older occurred in high-income countries, leading to 470,000 hospitalizations and 33,000 in-hospital deaths.^(6,7)^ In Brazil, according to the Influenza Syndrome Surveillance Report of November 21, 2023, the lethality associated with severe acute respiratory syndrome (SARS) due to RSV in adults was approximately 19% in this same year.^(8)^ The aim of a Brazilian retrospective study was to analyze the frequency rates and viral loads of RSV infections in different cohorts of patients and age groups over an eight-year period in a university hospital in São Paulo, and 1,380 immunocompetent (IC) and immunosuppressed (IS) patients with acute respiratory tract infections were analyzed. The IC group included patients with chronic heart disease, beneficiaries of primary care services and a subgroup with suspected SARS caused by the Influenza A (H1N1) virus. Respiratory samples with RSV detection and viral load quantification by real-time polymerase chain reaction (RT-PCR) were collected between February 2005 and October 2013. The overall rate of RSV infection was 17.3%, with higher rates in children (23.9%) compared to adults (12.9%), particularly in children under 2 years of age (28.2 %). Adults with heart disease had a significantly higher frequency rate (27.83%) than the SARS-H1N1 (2.65%) and IS (15.16%) subgroups, and the hospitalization rate was higher among adults under 65 years of age. Hospitalized patients had significantly higher RSV viral loads (7.34 ± 1.9) than outpatients (4.38 ± 1.89). Bone marrow transplant older adult patients also had significantly higher viral loads (7.57 ± 2.41) than younger adults (5.12 ± 1.87).^(9)^

In the USA, RSV is one of the main causes of hospitalization and mortality in older adults over 65 years of age. In 2022, according to the National Foundation for Infectious Diseases (NFID),^(10)^ around 60,000-160,000 hospital admissions and 6,000-10,000 deaths related to RSV were recorded. Compared to the other age group most affected by this virus, of children under 5 years, with 58,000-80,000 hospitalizations and 100-300 deaths, we notice a substantially higher incidence of hospitalizations and mortality due to RSV in older adults than in children.^(10)^ Note that the impact of the disease on older adults generates important healthcare costs for assisting infected patients.^(2)^

## What is the negative impact of respiratory diseases caused by RSV on adults?

To characterize the severity associated with RSV and the need to indicate vaccination to prevent the infection and its severe forms in adults over 60 years of age, the Center for Disease Control and Prevention (CDC)^(11)^ evaluated 5,784 hospitalized adults over 60 years of age with acute respiratory disease and laboratory-confirmed RSV, SARS-CoV-2 or influenza infection. From February 1, 2022 to May 31, 2023, patients of 25 hospitals in 20 US states were prospectively enrolled. Patients hospitalized with RSV were more likely to receive standard flow oxygen and be admitted to the ICU than those hospitalized for COVID-19 or influenza. Patients hospitalized for RSV were more likely to receive non-invasive ventilation or to die compared to patients hospitalized with influenza (adjusted odds ratio: 2.08; 95% confidence interval [CI]: 1.33-3.26). Although RSV was less common among hospitalized older adults, it was associated with more severe conditions compared to patients with COVID-19 or influenza. It is important to consider the high severity of the disease in older adults hospitalized with RSV when making shared clinical decisions regarding the recommendation of vaccination against RSV.^(11)^

## How are cases of respiratory infections recorded in Brazil?

In Brazil, the Influenza/COVID-19 Surveillance System captures cases in an integrated manner through the adopted definitions of flu syndrome and SARS. The clinical samples collected are also tested for RSV to make differential diagnosis between influenza and SARS-CoV-2. Thus, potential information is currently obtained to understand the epidemiological situation and the seasonal pattern of diseases caused by RSV in the country. With the SARS-CoV-2 pandemic, the InfoGripe system monitors SARS notification data in Brazil using the Sivep-gripe system of the Health Surveillance Secretariat of the Ministry of Health (SVS/MS) as data source, generating situation alerts based on the historical pattern of each region analyzed.^(12)^

From 2013 to 2016, 245 deaths from RSV were recorded in Brazil (61 in 2013, 53 in 2014, 50 in 2015 and 81 in 2016), which represents an average proportion of 2.6% of confirmed RSV cases (3.2% in 2013, 2.6% in 2014, 2.1% in 2015 and 2.6% in 2016). The age group of infants under 6 months of age had the highest number of deaths, except for the year 2016, when the highest number was in individuals aged 65 or over. However, the higher proportions of confirmed RSV cases resulting in death were observed among individuals aged 65 or over in all years studied (14.5% in 2013, 15.6% in 2014, 19% in 2015 and 22.5% in 2016).^(2)^

## What is the seasonality of RSV in Brazil?

When characterizing the seasonality of RSV in Brazil in the pre-pandemic period, the annual cycle reached the peak of positive cases for RSV in May. The circulation of RSV in Brazil begins in the states closer to the Equator and continues to the southern region of the country. In the Northeast and Southeast regions, peaks in cases were observed between April and May. In the South region, the peak occurred between June and July. The state of Rio Grande do Sul, further south, presented the last peak in cases.^(2)^

After the pandemic, until 2022-2023, the seasonality of RSV in Brazil and the world underwent changes. For example, until epidemiological week 24 of 2023, according to the São Paulo State Influenza Report,^(13)^ an increase in the number of SARS cases due to RSV was reported in the pandemic and post-pandemic period compared to the pre-pandemic period, as well as an increasing trend in the number of cases in the last months of 2021 and 2022, indicating a possible modification in the seasonal pattern of case occurrence after the COVID-19 pandemic.^(14)^

## What are the target populations for protection with RSV vaccination?

Today, there are different protection strategies according to the main target populations: maternal, young infants and older adults over 60 years of age. As RSV is rarely tested among women with respiratory tract infections, particularly during the influenza season, substantial uncertainties about the true prevalence of RSV infections remain.^(4)^ Interest in maternal infections and complications has recently been raised by the strategy of immunization against RSV in pregnant women with the aim to protect newborns during the first months of life. Future health technology assessments of maternal vaccination strategies will require a preventative and detailed definition of the burden of the disease and potential outcomes of vaccinating pregnant women with the primary objective of protecting the young infant through the transfer of antibodies during pregnancy.^(4)^

In this regard, maternal vaccination strategies appear particularly attractive, as transplacental transfer of neutralizing antibodies is well documented even in RSV infections and high maternal antibody titers have shown to be able to reduce the risk of childhood infections, particularly in early weeks of life. Pregnant women are at higher risk of serious disease and mortality due to infection with respiratory viruses, which represents an additional justification for their vaccination against RSV. Some studies have suggested that RSV infection during pregnancy may increase the risk of premature birth by cesarean section, and lead to higher rates of adverse pregnancy outcomes. While we await the results of large randomized clinical trials on maternal vaccination performed in our country, an updated synthesis of the literature is needed to determine whether or not RSV infection can be recognized as a rare occurrence in pregnant women and if the available evidence confirms or not that RSV infections in pregnancy are associated with more serious outcomes for mothers and children.^(4)^

Immunosenescence in adults over 65 years of age and the presence of additional comorbidities may compromise responses to vaccination and the assessment of efficacy. This population may benefit from inactivated vaccines with or without adjuvant systems, as studies have shown that booster doses and maintaining sufficiently high titers of neutralizing antibodies can provide protection against serious diseases. Live attenuated vaccines will probably not be effective in this age group, as pre-existing immunity would possibly prevent adequate viral replication and, therefore, an adequate immunogenicity response.^(15)^

## What are the molecular characteristics and serotypes of RSV?

The RSV is a pneumovirus of the *Paramyxoviridae* family. The viral genome consists of 10 genes that encode 11 proteins. The virus contains three encoded transmembrane surface glycoproteins: fusion (F), binding (G), the small hydrophobic SH protein and also a non-glycosylated matrix protein (M). Glycoproteins F and G induce most neutralizing antibody responses to infection. Glycoprotein G is a receptor for cell adhesion. The protein F induces fusion in cell cultures, from which the virus derived its name, and is probably responsible for the adhesion and dissemination of the virus in host cells.^(2)^

Given its essential role in viral entry into the host cell and because it is highly conserved between subtypes A and B, the protein F is the preferred target for vaccines. This protein induces the production of high-potency neutralizing antibodies in its two conformations: pre- and post-fusion (more than 90% are directed against this protein). These features of the protein F have revolutionized the field of RSV biology. The isolated RSV serotypes are divided into two subtypes based on antigenic and genetic characteristics: A and B. They can circulate together in the same season or alternate each year, although there is no consensus on differences in the severity of diseases caused by these two types.^(2)^

## How to diagnose infections caused by RSV?

Although the literature documents the increasing impact of RSV among adults, barriers to identify this virus may result in an underestimation of the problem. As the clinical characteristics of RSV infection are not specific in adults, it is challenging to distinguish it from other common respiratory viral infections.^(1)^

Therefore, laboratory confirmation is necessary to confidently establish a diagnosis of RSV infection. Even so, compared to infants and young children, virus titers in respiratory disease secretions in adults are lower and the duration of shedding is shorter, increasing the difficulty of identifying RSV in this population. During the acute phase of the disease, as the upper respiratory tract is also infected, the virus can be recovered in nasopharyngeal secretions by RT-PCR, immunofluorescence, antigen detection by enzyme immunoassays or cell culture.^(2)^ However, these tests are considered of low sensitivity in adults.^(1)^

## What are the strategies for preventing RSV infections in infants?

The suggested approach to protect young infants from severe RSV infections is passive immunization through monoclonal antibodies and/or maternal vaccination.^(15,16)^ The currently recommended prophylaxis to prevent severe forms of RSV infections in at-risk children is the monthly administration of palivizumab in newborns under 29^(17)^ or 32 weeks of gestational age during the first year of life.^(18,19)^ This monoclonal antibody presents neutralizing and fusion inhibitory activity against this virus. For its rational and efficient use, availability must be scheduled in accordance with timely surveillance data and virus circulation patterns. The first dose should be administered one month before the beginning of the RSV seasonality period with up to five sequential doses administered monthly during this period.^(2)^ Children with congenital heart disease with hemodynamic repercussions and chronic lung disease due to prematurity should also receive prophylaxis until the second year of life.^(18,19)^

Nirsevimab, a long-acting monoclonal antibody was recently approved in Brazil.^(20)^ A single dose is indicated for all children under 1 year of age (universal use) during the first season of RSV with administration prior to or during the season of greatest RSV circulation.^(10)^

In recent years, advances in understanding the biology of RSV have resulted in an increase in studies seeking to develop active immunization (prophylactic vaccines) against this virus. The development of high-quality, safe, and effective RSV preventive interventions for global use includes:^(10)^

- Maternal immunization to prevent RSV infections in infants under 6 months of age.

Efficacy and safety data from pregnancy vaccine studies were presented in a double-blind, randomized study of pregnant women between 24 and 36 weeks of gestation who received a single 120 μg intramuscular dose of a bivalent prefusion vaccine candidate (RSV A and B) based on protein F (RSVpreF) (3,682 participants) or placebo (3,676 participants). More than 7,000 pregnant women and their newborns who received the RSVpreF vaccine or placebo were evaluated. The two primary efficacy outcomes were RSV-associated severe respiratory tract disease with medical care and RSV-associated lower respiratory tract disease with medical care in infants within 90, 120, 150, and 180 days after birth. In the analysis, severe lower respiratory tract disease with medical care occurred within 90 days of birth, with vaccine efficacy of 81.8% (95% CI: 40-96); and 180 days after birth, vaccine efficacy was 69.4% (95% CI: 44.3-84.1). No safety signals were detected in maternal participants or in infants and children up to 24 months of age. The vaccine group and the placebo group reported similar incidences of adverse events within one month after dosing (13.1%) or within one month after birth (34.5%). Although not statistically significant, there was a slight increase in premature births and the development of gestational hypertensive disease in the vaccine group, with no causal relationship with immunization. Therefore, the RSVpreF vaccine administered during pregnancy was effective in preventing serious RSV-associated lower respiratory tract disease with medical care in infants, and to date without safety concerns.^(21)^

## What are the strategies for preventing RSV infections in adults?

- Immunization of adults (over 60 years of age) to prevent RSV infections.

Placebo-controlled, randomized clinical trials were carried out with two vaccines; one already approved by the National Health Surveillance Agency – Anvisa (Arexvy^®^) and the other awaiting evaluation (Abrysvo^®^). Regarding the first vaccine (Arexvy^®^), adults aged 60 years and older received a single dose of the AS01E-adjuvanted prefusion RSV F protein-based vaccine (RSVPreF3 OA) or placebo. The primary objective of the study was to demonstrate the efficacy of one dose of the RSVPreF3 OA vaccine against RSV-associated lower respiratory tract disease confirmed by RT-PCR. Secondary outcomes included assessment of the efficacy against RSV-associated severe lower respiratory tract disease, RSV-associated acute respiratory infection, and lower respiratory tract disease according to RSV subtype (A or B), participant age group, presence or absence of comorbidities and frailty status at the beginning of the study. The comorbidities of interest assessed were chronic lung diseases such as COPD and asthma, heart diseases such as congestive heart failure and coronary disease, types 1 and 2 diabetes, and liver and kidney failure.^(22)^

Vaccine efficacy against RSV-associated lower respiratory tract disease confirmed by RT-PCR was 82.6% (95% CI: 58-94%). Vaccine efficacy against RSV-associated severe lower respiratory tract disease was 94.1% (95% CI: 62-99%) and against RSV-associated acute respiratory infection, 71.7% (95% CI: 56-82%). The effectiveness of the vaccine for those with at least one comorbidity was 94.6% (95% CI: 66-99%). Vaccine efficacy was similar against RSV subtypes A and B for both RSV-associated lower respiratory tract disease (84.6% [subtype A] and 80.9% [subtype B]) and RSV-associated acute respiratory infection (71.9% [subtype A] and 70.6% [subtype B]). Regarding safety, the RSVPreF3 OA vaccine was more reactogenic than placebo, but most adverse events were transient (mild to moderate severity) with an average duration of one to two days. The incidences of serious adverse events and potential immune-mediated diseases were similar in both groups. Therefore, the vaccine demonstrated an acceptable safety profile and prevented acute respiratory infection, lower respiratory tract disease, and RSV-associated severe disease in adults aged 60 years and older, regardless of RSV subtype and the presence of underlying coexisting conditions.^(22)^

Still in relation to immunization in older adults regarding the efficacy and safety data of the vaccine studies being analyzed, we have the results of a randomized placebo-controlled clinical trial in adults over 60 years of age. Participants received a single intramuscular injection of RSVpreF vaccine at a dose of 120 μg (RSV subgroups A and B, 60 μg each) or placebo. The two primary outcomes were vaccine efficacy against RSV-associated seasonal lower respiratory tract diseases with at least two or three signs or symptoms. The secondary outcome was vaccine efficacy against RSV-associated acute respiratory diseases. This vaccine (Abrysvo^®^) for RSV-associated lower respiratory tract disease with at least two signs or symptoms showed efficacy of 66.7% (95% CI 28-85). Vaccine efficacy for RSV-associated lower respiratory tract disease with at least three signs or symptoms was 85.7% (95% CI: 32-72). RSV-associated acute respiratory disease showed efficacy of 62.1% (95% CI: 37-77). Regarding safety, the incidence of local reactions was higher with the vaccine (12%) than with placebo (7%) and the incidences of systemic events were similar (27% and 26%, respectively). Similar rates of adverse events were reported up to one month after injection (vaccine: 9.0%; placebo: 8.5%). Serious or life-threatening adverse events were reported in 0.5% of those who received the vaccine and in 0.4% of those who received placebo. Serious adverse events were reported in 2.3% of participants in each group by the data cutoff date. Therefore, the vaccine prevented RSV-associated lower respiratory tract diseases and RSV-associated acute respiratory diseases in adults (over 60 years of age) without safety concerns.^(23,24)^

In a recent publication, a randomized, double-blind, placebo-controlled, phase 2-3 RSV vaccine study of adults aged 60 years and older is underway. Participants received one dose of the vaccine candidate (mRNA-1345 [50 μg]) or a placebo dose. The final objective was to evaluate the effectiveness and safety in preventing RSV-associated acute respiratory disease. As a result, a single dose of the mRNA-1345 vaccine was safe and showed a lower incidence of RSV-associated lower respiratory tract diseases compared to placebo among adults aged 60 years and older.^(25,26)^

## How are the analyzes of Regulatory Agencies in the world and in Brazil?

On August 21, 2023, the United States Food and Drug Administration (FDA) approved a vaccine against RSV for use in pregnant women to prevent RSV-associated lower respiratory tract diseases and its severe form in babies from birth to 6 months of age (Abrysvo^®^). The vaccine was approved for use between 32 and 36 weeks of gestational age, administered as a single-dose intramuscular injection.^(22)^ The European Union, through the European Medicines Agency (EMA), also authorized the same vaccine for use in pregnant women between 24 and 36 weeks of gestation in a single dose intramuscularly.^(23)^ In Brazil, this vaccine was submitted for approval and at the time of preparing this Febrasgo Position Statement (FPS), it is under analysis by Anvisa.

Approvals from Regulatory Agencies:

- The FDA has approved two RSV vaccines for adults:
  – Arexvy^®^ GSK on May 3, 2023 for older adults aged 60 and over;^(27)^
  – Abrysvo^®^ Pfizer on May 31, 2023 for older adults aged 60 and over.^(28)^

On June 21, 2023, the CDC Advisory Committee on Immunization Practices recommended RSV vaccination for adults over the age of 60 years offered to individual adults, recommending the clinical decision to be shared.^(3)^

On August 21, 2023, the United States agency FDA approved a vaccine against RSV for use in pregnant women to prevent RSV-associated lower respiratory tract diseases and its severe form in babies from birth to 6 months of age (Abrysvo^®^).^(22)^ The vaccine was approved for use between 32 and 36 weeks of gestational age, administered as a single-dose intramuscular injection.^(28)^

The European Union, through the EMA, also authorized the same vaccine for use in pregnant women between 24 and 36 weeks of gestation in a single dose intramuscularly.^(29)^

In Brazil, this vaccine was submitted for approval and at the time of preparing this FPS, it was under analysis by the Anvisa.^(30)^

## Final considerations

The RSV infection is an important virus in extreme age groups, infants, children, older adults and high-risk adults, with a negative impact on health similar to that of the non-pandemic influenza A. In this special population, the vaccination rate against influenza is high and with the recent approval of the RSV vaccine for older adults in our country and the possible approval for pregnant women, health professionals will be able to offer the benefits of protection against this infection.

## National Commission Specialized in vaccines of the Brazilian Federation of Gynecology and Obstetrics Associations (Febrasgo)

### President:

Cecilia Maria Roteli Martins

### Vice-president:

Nilma Antas Neves

### Secretary:

Susana Cristina Aidé Viviani Fialho

### Members:

André Luis Ferreira Santos
  Angelina Farias Maia
  Fabiola Zoppas Fridman
  Giuliane Jesus Lajos
  Isabella de Assis Martins Ballalai
  Juarez Cunha
  Julio Cesar Teixeira
  Manoel Afonso Guimaraes Goncalves
  Marcia Marly Winck Yamamoto De Medeiros
  Renata Robial
  Renato de Ávila Kfouri
  Valentino Antonio Magno
